# The Association Between Glucose Intolerance and Longitudinal Fetal Growth in Nulliparas

**DOI:** 10.1097/og9.0000000000000213

**Published:** 2026-10-27

**Authors:** Alexandra Mahdasian-Miller, Armando Peña, David Haas, Maisa Feghali, Lynn Yee, Jessica Wilder, Nathan Blue, Celeste Durnwald, Jonathan Steller, Christina M. Scifres

**Affiliations:** Department of Obstetrics & Gynecology, Vanderbilt University Medical Center, Nashville, Tennessee; Department of Health & Wellness Design, School of Public Health, Indiana University, Bloomington, Indiana; Department of Obstetrics & Gynecology, Indiana University School of Medicine, Indianapolis, Indiana; Department of Obstetrics & Gynecology, University of Pittsburgh, Pittsburgh, Pennsylvania; Department of Obstetrics & Gynecology, Northwestern University Feinberg School of Medicine, Chicago, Illinois; Department of Obstetrics & Gynecology, Case Western Reserve University, Cleveland, Ohio; Department of Obstetrics & Gynecology, University of Utah Health, Salt Lake City, Utah; Department of Obstetrics & Gynecology, University of Pennsylvania Perelman School of Medicine, Philadelphia, Pennsylvania; Department of Obstetrics & Gynecology, University of California Irvine School of Medicine, Irvine, California; and Department of Obstetrics & Gynecology, Indiana University School of Medicine, and Regenstrief Institute, Indianapolis, Indiana.

## Abstract

**OBJECTIVE:**

To evaluate fetal growth patterns and birth weight among individuals with normoglycemia, mild glucose intolerance, and gestational diabetes mellitus (GDM) from midgestation until delivery.

**METHODS:**

This is a secondary analysis of the NuMoM2b (Nulliparous Pregnancy Outcomes Study: Monitoring Mothers-to-Be) prospective cohort of nulliparous individuals with singleton gestations. Maternal glucose tolerance was categorized as normal, mild glucose intolerance (one abnormal Carpenter–Coustan value), or GDM. Blinded ultrasounds at 16–22 weeks and 22–29 weeks provided fetal biometry *z* scores; suspected large for gestational age (LGA) was defined as an abdominal circumference or estimated fetal weight above the 90th percentile on ultrasound; LGA was confirmed as birth weight above the 90th percentile for gestational age and sex. Logistic regression and covariance pattern models assessed the association between glycemic status and LGA growth across time points.

**RESULTS:**

Among 7,010 included participants, 175 (2.5%) had mild glucose intolerance, and 290 (4.1%) had GDM. Fetal biometry was similar across groups at 16–22 weeks. By 22–29 weeks, GDM was associated with accelerated fetal growth *z* scores, and both mild glucose intolerance and GDM were associated with increased odds of abdominal circumference above the 90th percentile (adjusted odds ratio [AOR] 1.64, 95% CI, 1.08–2.50 and AOR 1.54, 95% CI, 1.10–2.16, respectively). By delivery, mild glucose intolerance carried a twofold increased risk for LGA birth weight (AOR 2.00, 95% CI, 1.29–3.10), whereas the LGA risk in those with GDM was similar to that in normoglycemic individuals (AOR 1.34, 95% CI, 0.91–1.98).

**CONCLUSION:**

Fetal overgrowth associated with glucose intolerance emerges around the standard 24- to 28-week GDM screening window. Individuals with GDM had an LGA risk similar to those with normal glucose tolerance, consistent with the increased surveillance and clinical management associated with a GDM diagnosis attenuating the trajectory of excess fetal growth. Whether treatment of mild glucose intolerance can reduce LGA risk remains to be determined.

**CLINICAL TRIAL REGISTRATION:**

ClinicalTrials.gov, NCT01322529.

## Abstract 2

Fetal overgrowth associated with glucose intolerance emerges around the 24- to 28-week testing window, and current gestational diabetes mellitus treatment appears to modify this trajectory.

The prevalence of gestational diabetes mellitus (GDM) is increasing,^1^ which is associated with adverse outcomes, including large-for-gestational-age (LGA) birth weight.^2^ Large-for-gestational-age birth weight is associated with short- and long-term morbidities, including offspring risk of obesity, diabetes, and cardiovascular disease.^3^ Treatment of GDM identified between 24 and 28 weeks reduces LGA birth weight and other morbidity,^4,5^ supporting recommendations for universal GDM testing at this gestational window.^2,6,7^ Individuals with a single abnormal Carpenter–Coustan value typically do not receive treatment yet remain at increased risk for LGA birth weight compared with those with normal glucose tolerance or a formal GDM diagnosis.^8^

Classically, GDM is characterized as a late pregnancy condition driven by escalating physiologic insulin resistance.^9^ However, many individuals enter pregnancy with preexisting dysglycemia; in the United States, roughly 10.0% of individuals with diabetes remain undiagnosed,^10^ and up to 26.0% of reproductive-aged women have prediabetes.^11^ Randomized clinical trials evaluating whether earlier GDM screening improves perinatal outcomes have failed to demonstrate a reduction in LGA birth weight with treatment initiated before 24 weeks.^12,13^ Characterizing the timing and trajectory of fetal overgrowth across the spectrum of glucose intolerance may help explain why certain interventions succeed or fail and inform targeted screening and treatment strategies.

Given the lack of consensus on optimal GDM diagnosis and treatment timing, we evaluated ultrasound biometry at 16–22 weeks, 22–29 weeks, and delivery among pregnancies with normal glucose tolerance, mild glucose intolerance, and GDM, hypothesizing that fetal growth trajectories would differ across glycemic groups.

## METHODS

Data for this study are derived from the NuMoM2b (Nulliparous Pregnancy Outcomes Study: Monitoring Mothers-to-Be) study. The NuMoM2b study included nulliparous individuals with no prior pregnancy who progressed beyond 20 weeks of gestation and carried singleton gestations. Individuals were enrolled at multiple geographically diverse clinical sites between October 2010 and September 2013. Trained research personnel collected data at three study visits (visit 1 between 6 0/7 and 13 6/7 weeks, visit 2 between 16 0/7 and 21 6/7 weeks, and visit 3 between 22 0/7 and 29 6/7 weeks; each study visit occurred a minimum of 4 weeks after the prior visit). After delivery, trained and certified chart abstractors reviewed the medical records of all participants and recorded final birth outcomes. The local IRBs approved the NuMoM2b study, and all individuals provided written informed consent before participation. Full details of the study protocol have previously been published.^14^

For this study, pregnancy dating was performed using ultrasound before 13 weeks of gestation. Trained research personnel collected data during the three study visits. These analyses focus on the fetal biometry performed at study visits 2 and 3. The clinical team and patient were blinded to the data from both ultrasound evaluations unless a major structural malformation, hydrops, fetal death, an estimated fetal weight below the 5th percentile, or oligohydramnios (maximum vertical pocket less than 2 cm or amniotic fluid index less than 5 cm) was identified. The head circumference and abdominal circumference were measured with the ellipse function on the machine at the standard anatomic sites, and the femur length was obtained as a linear measurement. The estimated fetal weight was calculated with the Hadlock formula using head circumference, abdominal circumference, and femur length.^15^ To allow for variation in the exact timing of the ultrasounds at visit 2 and visit 3, *z* scores for ultrasound-derived estimated fetal weight, head circumference, abdominal circumference, and femur length were calculated from the distribution of measurements within the dataset. Suspected LGA for any given ultrasound measurement was defined as an abdominal circumference or estimated fetal weight measurement above the 90th percentile for gestational age and sex. Sex- and gestational age–corrected birth weight *z* scores were calculated with a population-based U.S. reference, and LGA was defined as a birth weight above the 90th percentile.^16^

All pregnant individuals without a diagnosis of pregestational diabetes were offered GDM testing as part of routine clinical care. This analysis included participants who underwent GDM screening after 24 weeks of gestation with a two-step approach: a 50-g oral glucose challenge test (GCT) followed by a 100 g oral glucose tolerance test (OGTT) if the initial GCT exceeded 130–140 mg/dL (7.2–7.8 mmol/L). Gestational diabetes was diagnosed if two or more values met or exceeded the Carpenter–Coustan thresholds: fasting 95 mg/dL (5.3 mmol/L) or higher, 1 hour 180 mg/dL (10.0 mmol/L) or higher, 2 hour 155 mg/dL (8.6 mmol/L) or higher, or 3 hour 140 mg/dL (7.8 mmol/L) or higher. Participants with a single abnormal value were classified as having mild glucose intolerance and did not meet formal criteria for GDM diagnosis or treatment; any diabetes medication use in this group is described in the Results.

We excluded NuMoM2b participants who did not undergo standard GDM screening or diagnosis with either a 50-g GCT or 100-g OGTT and those who had GDM testing performed before 24 weeks of gestation (n=1,244). We also excluded participants with a known diagnosis of pregestational diabetes or an hemoglobin A_1C_ exceeding 6.5% at the first study visit if measured. Routine early-pregnancy screening for overt pregestational diabetes was not a standardized component of the NuMoM2b protocol. In addition, we excluded pregnancies complicated by stillbirth, fetal aneuploidy, or a significant structural anomaly. Lastly, participants with missing ultrasound data from visits 2 or 3 were omitted.

Baseline demographics were compared among individuals with normal glucose tolerance, mild glucose intolerance, and GDM with χ^2^ tests or one-way analysis of variance as appropriate. To assess the association between maternal glycemia and extremes of fetal growth, we considered whether exposure to either normoglycemia, mild glucose intolerance, or GDM was associated with either an abdominal circumference or estimated fetal weight above the 90th percentile at visit 2 (16–22 weeks) or visit 3 (22–30 weeks) or a birth weight above the 90th percentile. Covariates included in the multivariable logistic regression analyses were selected according to baseline demographics that differed among groups with values of *P*<.05. Race and ethnicity were self-reported by participants using prespecified categories in the NuMoM2b data collection instruments and were included in multivariable models as a marker for the structural racism that may affect health outcomes. These statistical analyses were completed with Stata 18.

Covariance pattern models were used to assess differences in ultrasound biometry *z* scores and estimated fetal weight and birth weight *z* scores over time between groups. Covariance pattern models are based on the covariance structure of the data. For each analysis, we identified the most parsimonious model (ie, the covariance structure with model convergence and the lowest Bayesian Information Criteria without any covariate adjustments. We applied compound-symmetry covariance structures for the femur length, abdominal circumference, and head circumference models and an unstructured covariance structure for the estimated fetal weight models. Adjusted models included maternal age, race, insurance status, body mass index (BMI) category, and chronic hypertension as covariates on the intercept and slope. An α level of .05 was used for all comparisons; given the exploratory nature of this analysis and the complexity of the models, no adjustment for multiple comparisons was made. SAS 9.4 was used to run covariance pattern models. No artificial intelligence tools were used in the writing, analysis, or preparation of this manuscript.

## RESULTS

The parent NuMoM2b study enrolled 10,038 nulliparous individuals between October 2010 and May 2013. Our final analytic cohort comprised 7,010 participants (69.8%, Fig. 1). Primary reasons for exclusion included pregestational diabetes (or unknown status), GDM testing performed before 24 weeks of gestation, and incomplete ultrasound data from either visit 2 or 3. Among the 7,010 included participants, 175 (2.5%) had mild glucose intolerance, and 290 (4.1%) had a diagnosis of GDM. Baseline maternal characteristics differed across the three groups (Table 1). Individuals with mild glucose intolerance of GDM were generally older and had higher rates of maternal obesity and chronic hypertension compared with the normoglycemic group. A lower proportion of participants identifying as White was observed in the GDM group. Maternal insurance status differed significantly across groups, with the highest prevalence of private insurance among individuals with mild glucose intolerance. The mean gestational age for visits 2 (18.8±1.5 weeks vs 18.7±1.4 weeks vs 18.9±1.4 weeks, *P*=.39) and 3 (27.2±1.7 weeks vs 27.4±1.6 weeks vs 27.3±1.8 weeks, *P*=.21) were similar among those with normal glucose tolerance, mild glucose intolerance, and GDM.

**Fig. 1.**
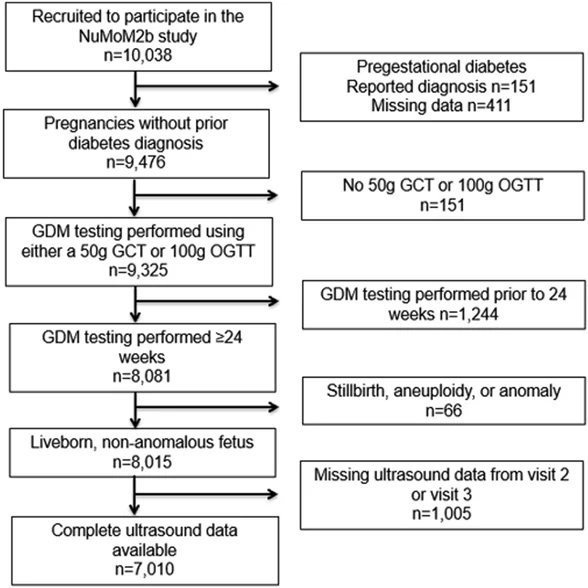
Derivation of the study cohort. NuMom2b, Nulliparous Pregnancy Outcomes Study: Monitoring Mothers-to-Be; GCT, glucose challenge test; OGTT, oral glucose tolerance test; GDM, gestational diabetes mellitus.

*Mahdasian-Miller. Glucose Intolerance and Fetal Growth. O&G Open 2026*.

**Table 1.** Maternal Demographics and Delivery Characteristics

	No GDM (n=6,545)	Mild Glucose Intolerance (n=175)	GDM (n=290)	*P*
Maternal demographics				
Maternal age (y)	27.1±5.6	29.3±5.1	29.5±5.7	<.001
Race (n=7,009)				<.001
Asian	262 (4.0)	13 (7.4)	35 (12.1)	
Black	829 (12.7)	24 (13.7)	51 (17.6)	
Hispanic	1,026 (15.7)	15 (8.6)	23 (7.9)	
White	4,117 (62.9)	114 (65.1)	167 (57.6)	
None of the above	310 (4.7)	9 (5.1)	14 (4.8)	
Education (n=7,007)				.74
High school or less	2,309 (35.3)	57 (32.6)	100 (34.6)	
At least some college	4,234 (64.7)	118 (67.4)	189 (65.4)	
Insurance (n=6,968)				<.001
Private	3,526 (54.2)	125 (71.4)	162 (55.9)	
Public	1,629 (25.0)	34 (19.4)	74 (25.5)	
Other	1,348 (20.7)	16 (9.1)	54 (18.6)	
Early pregnancy BMI category (n=6,897)				<.001
Underweight	593 (9.2)	12 (6.9)	16 (5.6)	
Normal weight	3,058 (47.5)	71 (41.0)	86 (30.3)	
Overweight	1,649 (25.6)	39 (22.5)	73 (25.7)	
Obese	1,140 (17.7)	51 (29.5)	109 (38.4)	
Chronic hypertension (n=7,002)	106 (1.6)	8 (4.6)	15 (5.2)	<.001
Weight gain (n=6,827)	34.9±15.2	35.0±14.3	29.2±15.9	<.001
GDM testing (wk)	27.7±5.9	27.7±2.9	27.6±3.6	.93
Tobacco use (n=7,006)	1,069 (16.3)	35 (20.0)	58 (20.0)	.12
Gestational age at visit 2 (16–22 wk) (n=6,973) (wk)	18.8±1.5	18.7±1.44	18.9±1.4	.39
Gestational age at visit 3 (22–30 wk) (n=6,975) (wk)	27.2±1.7	27.4±1.6	27.3±1.8	.21
Delivery characteristics				
Gestational age at delivery (n=7,009) (wk)	39.0±1.7	38.7±2.1	38.4±1.8	<.001
Preterm birth (n=7,009)	410 (6.3)	17 (9.7)	31 (10.7)	.003
Birth weight (n=6,991) (g)	3,301±510	3,372±620	3,268±556	.11
Birth weight *z* score (n=6,985)	−0.11±0.93	0.21±1.02	0.01±1.00	<.001
Neonatal sex (n=6,998)				.86
Female	3,174 (48.6)	87 (49.7)	145 (50.0)	
Male	3,359 (51.4)	88 (50.3)	145 (50.0)	
Mode of delivery				<.001
Vaginal	4,834 (73.9)	95 (54.3)	192 (63.6)	
Cesarean	1,711 (26.1)	80 (45.7)	110 (36.4)	

GDM, gestational diabetes mellitus; BMI, body mass index.

Data are shown as n (%), mean±SD, or median (interquartile range). Data shown are complete unless the n available is noted.

Clinical management and delivery outcomes also varied by glycemic status. Individuals with GDM exhibited lower gestational weight gain. Individuals with mild glucose intolerance and those with GDM had lower gestational ages at delivery and higher rates of preterm birth than the normoglycemic reference group (Table 1). Of note, although absolute birth weights were similar across the three cohorts, the birth weight *z* score was highest among offspring of individuals with mild glucose intolerance. In addition, the incidence of cesarean delivery was significantly elevated in both the mild glucose intolerance and GDM groups. To characterize treatment patterns, we assessed the use of diabetes medications (including glyburide, metformin, and insulin). Diabetes medication use was documented in 20.34% of individuals with GDM. In the normoglycemic group, 0.9% of individuals reported using a diabetes medication during pregnancy. Among those with mild glucose intolerance, 6.3% received pharmacologic therapy (including three glyburide and seven metformin users), despite not meeting the formal diagnostic criteria for GDM. All seven metformin users also had a diagnosis of polycystic ovary syndrome.

We compared *z* score trajectories for femur length, abdominal circumference, and head circumference at visits 2 and 3. There were no differences in head circumference or femur length trajectories across the three groups (Fig. 2A and 2B) either before or after adjustment for covariates including maternal age, BMI, and insurance status. When evaluating abdominal circumference *z* scores, we observed no differences at visit 2. However, by visit 3, the abdominal circumference was significantly larger in pregnancies complicated by GDM than in those with normal glucose tolerance, both before and after adjustment for covariates (Fig. 2C). No statistically significant differences in abdominal circumference *z* scores were detected between the normoglycemic and mild glucose intolerance groups at either point (Fig. 2C). When we compared trajectories in *z* scores across visits, the most parsimonious models for femur length, abdominal circumference, and head circumference used compound symmetry covariance structures.

**Fig. 2.**
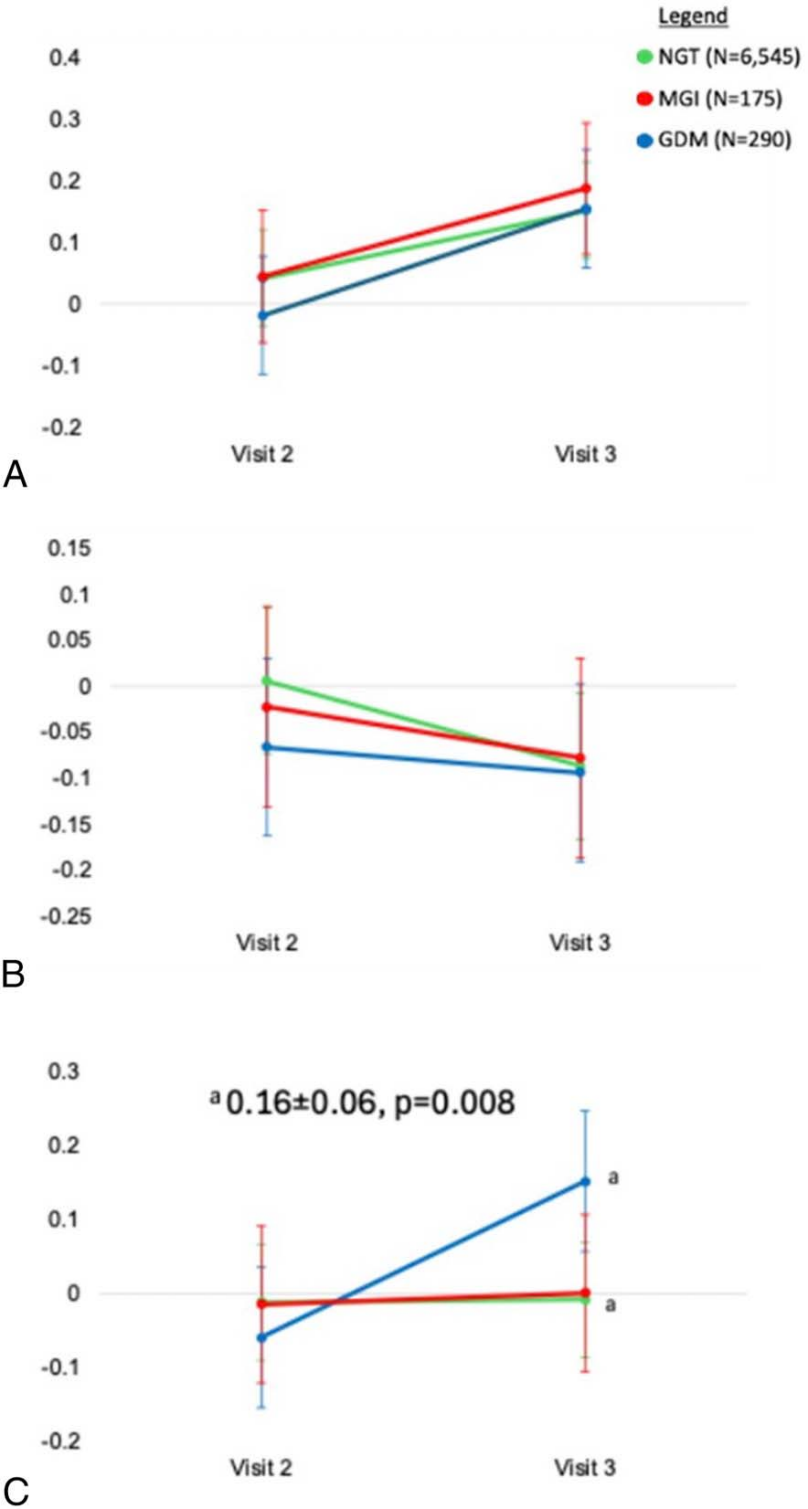
Adjusted covariance pattern model estimates of fetal biometry *z* score trajectories from visit 2 (16–22 weeks of gestation) to visit 3 (22–29 weeks of gestation) by maternal glycemic category. Femur length *z* score **(A)**, head circumference *z* score **(B)**, and abdominal circumference *z* score **(C)**. Models adjusted for maternal age, race, insurance status, body mass index category, and chronic hypertension. Significance markers denote pairwise comparisons with *P*<.05; all group estimates are shown. Data are shown as mean difference (Δ) ±SE. NGT, normal glucose tolerance; MGI, mild glucose intolerance; GDM, gestational diabetes mellitus.

*Mahdasian-Miller. Glucose Intolerance and Fetal Growth. O&G Open 2026*.

We next evaluated trajectories for estimated fetal weight and birth weight *z* scores from visits 2 and 3 to delivery using models fitted with an unstructured covariance structure. There were no differences among the three groups at visit 2 either before or after adjustment for covariates (Fig. 3A). By visit 3, GDM was associated with a higher estimated fetal weight *z* score than normal glucose tolerance, with no significant differences between those with normal glucose tolerance and those with mild untreated glucose intolerance (Fig. 3A). However, these results were no longer statistically significant after adjustment for covariates (Fig. 3B). By delivery, there were significant differences in birth weight *z* score among those with normal glucose tolerance, mild glucose intolerance, and GDM (Fig. 3A). However, after adjustment for covariates (age, race, insurance, BMI, and hypertension), the mild glucose intolerance group had a significantly higher birth weight *z* score compared with those with normal glucose tolerance or GDM (*P*<.001 vs normal, *P*=.023 vs GDM; Fig. 3B).

**Fig. 3.**
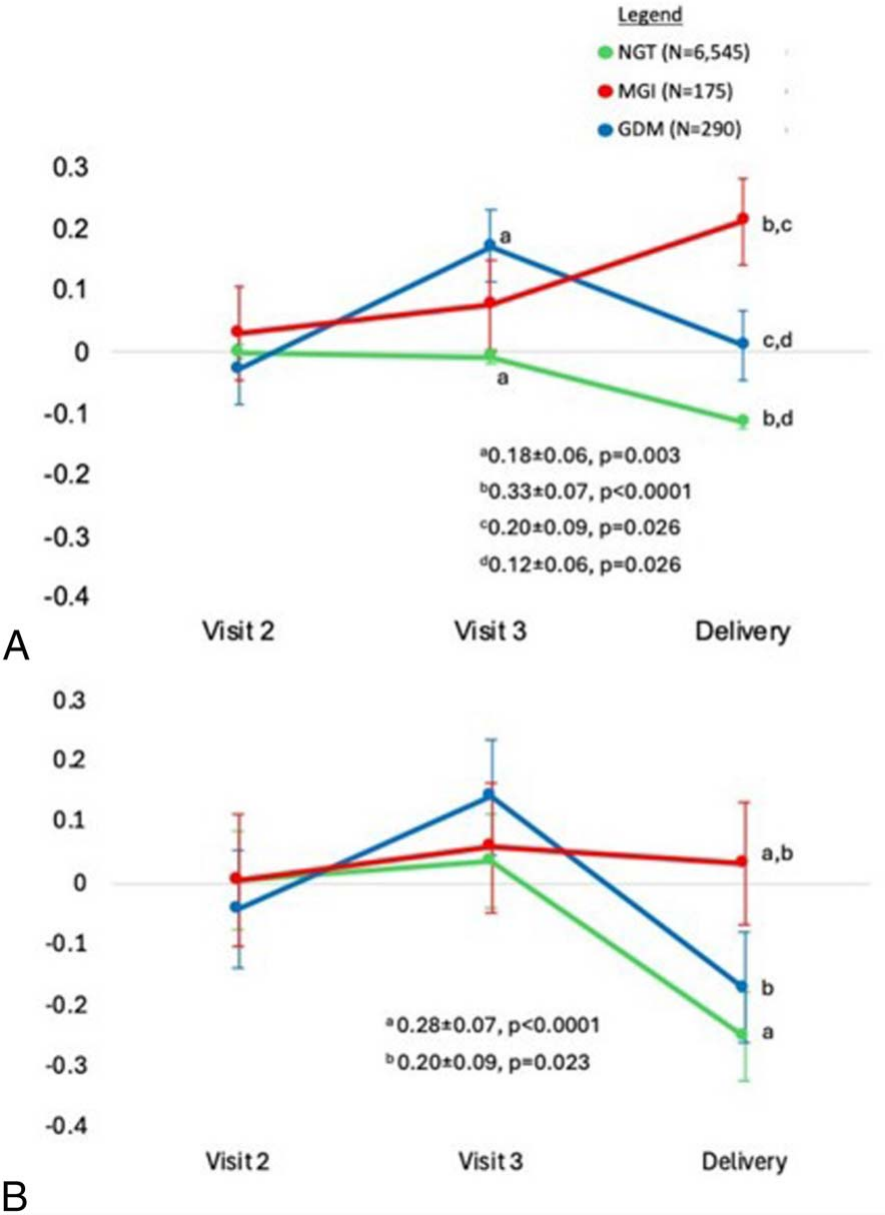
Covariance pattern model estimates of estimated fetal weight and birth weight *z* score trajectories from visit 2 (16–22 weeks of gestation) and visit 3 (22–29 weeks of gestation) to delivery by maternal glycemic category. Unadjusted estimates **(A)** and estimates adjusted for maternal age, race, insurance status, body mass index category, and chronic hypertension **(B)**. Significance markers denote pairwise comparisons with *P*<.05; all group estimates are shown. Data are shown as mean difference (Δ) ±SE. NGT, normal glucose tolerance; MGI, mild glucose intolerance; GDM, gestational diabetes mellitus.

*Mahdasian-Miller. Glucose Intolerance and Fetal Growth. O&G Open 2026*.

We also compared the prevalence of abdominal circumference and estimated fetal weight/birth weight above the 90th percentile (Table 2). At visit 2, there was a trend toward higher rates of abdominal circumference and estimated fetal weight above the 90th percentile in the GDM group, although this was not statistically significant after adjustment for BMI. By visit 3, however, there was a significantly increased likelihood of abdominal circumference above the 90th percentile in both the mild glucose intolerance and GDM groups (Table 2). Most strikingly, by the time of delivery, individuals with mild glucose intolerance were at a twofold increased risk for LGA birth weight (adjusted odds ratio 2.00, 95% CI, 1.29–3.10) compared with those with normal glucose tolerance. In contrast, individuals with GDM had an LGA risk similar to that of individuals with normal glucose tolerance (adjusted odds ratio 1.34, 95% CI, 0.91–1.98).

**Table 2.** Relationship Between Mild Glucose Tolerance, Gestational Diabetes, and Large-for-Gestational-Age Growth Patterns

Outcome	Growth Parameters Above the 90th Percentile				Unadjusted OR (95% CI)		Adjusted OR (95% CI)	
	Normal Glucose Tolerance (n=6,545)	Mild Glucose Intolerance (n=175)	GDM (n=290)	*P*	Mild Glucose Intolerance	GDM	Mild Glucose Intolerance	GDM
16–22 wk								
AC above the 90th percentile (n=6,943)	652 (10.1)	14 (8.1)	40 (13.8)	.08	0.79 (0.45–1.37)	1.44 (1.02–2.02)	0.73 (0.42–1.27)	1.34 (0.94–1.90)
EFW above the 90th percentile (n=6,943)	632 (9.8)	14 (8.1)	39 (13.5)	.08	0.81 (0.47–1.42)	1.44 (1.02–2.04)	0.75 (0.43–1.31)	1.31 (0.92–1.87)
22–30 wk								
AC above the 90th percentile (n=6,953)	618 (9.5)	29 (16.7)	47 (16.5)	<.001	1.90 (1.27–2.86)	1.88 (1.36–2.60)	1.64 (1.08–2.50)	1.54 (1.10–2.16)
EFW above the 90th percentile (n=6,953)	633 (9.8)	22 (12.6)	41 (14.2)	.02	1.34 (0.85–2.11)	1.56 (1.11–2.19)	1.17 (0.74–1.87)	1.30 (0.91–1.85)
Delivery								
Birth weight above the 90th percentile (n=7,004)	460 (7.0)	26 (14.9)	32 (11.0)	<.001	2.31 (1.50–3.54)	1.64 (1.12–2.40)	2.00 (1.29–3.10)	1.34 (0.91–1.98)

GDM, gestational diabetes mellitus; OR, odds ratio; AC, abdominal circumference; EFW, estimated fetal weight.

Normal glucose tolerance was used as the reference for the logistic regression models. Models adjusted for maternal BMI at visit 1, maternal age at visit 1, maternal race, insurance status, and chronic hypertension.

## DISCUSSION

Higher abdominal circumference *z* scores among individuals with GDM, together with increased rates of abdominal circumference above the 90th percentile in both the mild glucose intolerance and GDM groups at a mean gestational age of 27 weeks, suggest that fetal overgrowth associated with gestational glucose intolerance emerges around the standard 24- to 28-week GDM screening window. The similar LGA risk observed in the GDM group compared with normoglycemic individuals may reflect the effect of increased glycemic surveillance, dietary modification, and pharmacologic therapy when indicated—all components of clinical GDM management—on fetal growth trajectories. Although the observational nature of this study precludes direct conclusions about treatment efficacy, these findings suggest that if fetal overgrowth does not emerge until the standard screening window, this may provide context for understanding why prior randomized trials of early GDM treatment, which used differing diagnostic criteria and treatment timing, did not demonstrate a reduction in LGA birth weight.^12,13^

In this cohort, mild glucose intolerance was associated with a 2-fold increased risk for LGA birth weight. Notably, the association between mild glucose intolerance and elevated birth weight *z* score strengthened after adjustment for maternal age, race, insurance status, BMI, and chronic hypertension, suggesting that excess growth in this group reflects glucose-dependent pathways. The absence of clinical intervention in mild glucose intolerance, in contrast to recommended treatment in GDM, may also contribute to this divergence. Maternal glucose concentrations are positively associated with birth weight,^17^ and a single abnormal value on a 100-g, 3-hour OGTT is associated with increased LGA risk.^8^ The GDM diagnostic criteria vary internationally; broader criteria such as the one-step International Association for the Diabetes in Pregnancy Study Group approach increase GDM prevalence twofold to threefold without demonstrating a reduction in LGA birth weight,^18–22^ underscoring the unresolved question of whether individuals with a single abnormal value warrant clinical intervention.

Of note, 6.3% of individuals with mild glucose intolerance received oral diabetes medications despite not meeting formal GDM criteria, likely reflecting individualized clinical decision making or metformin use for other indications such as polycystic ovary syndrome. Treatment of some individuals in this group may have influenced the observed birth weight outcomes, although we expect any bias would be toward the null. The proportion of individuals with GDM receiving pharmacologic therapy (20.3%) is lower than contemporary national estimates, likely reflecting the high proportion of diet-treated GDM within this nulliparous cohort.

Our findings add to the literature evaluating growth trajectories before GDM diagnosis. Cohorts from the UK and the *Eunice Kennedy Shriver* National Institute of Child Health and Human Development Fetal Group Study-Singletons identified accelerated growth reaching significance between 24 and 28 weeks,^23–25^ whereas a South African cohort identified differences as early as 16–18 weeks.^26^ In contrast, studies from Norway, the Netherlands, Greece, and China reported no significant association between GDM and late-pregnancy growth trajectories.^27–30^ These discrepancies likely reflect differences in sample size, ultrasound frequency, and modeling methodologies.

A primary strength of this study is its prospective design within the NuMoM2b cohort, using standardized, repeated ultrasound biometry evaluations and detailed delivery outcomes. Blinding of ultrasound data reduced management bias based on estimated fetal size. Although limiting the analysis to nulliparous individuals may restrict generalizability, it eliminates confounding by maternal parity. The prevalence of GDM in this study was lower than contemporary national estimates, consistent with the nulliparous study population and the use of the two-step Carpenter–Coustan diagnostic criteria. Limitations include the lack of detailed GDM treatment information beyond medication use and the unavailability of glycemic data, including fasting glucose, mean blood sugars, and hemoglobin A_1C_, precluding direct assessment of glycemic control. Our analyses did not address other outcomes such as shoulder dystocia, birth injury, neonatal hypoglycemia, or hyperbilirubinemia or the potential long-term risk for childhood obesity in offspring. Lastly, given the exploratory nature of this analysis, no adjustment for multiple comparisons was performed; findings should be interpreted as hypothesis generating and confirmed in future prospective studies.

Our findings may provide context for understanding why early GDM treatment does not reduce LGA birth weight. However, important questions persist regarding optimal management of mild hyperglycemia in early pregnancy. Although the wide gestational age windows of our study visits preclude identification of when fetal growth divergence begins and the observational design limits conclusions about treatment effects, these findings suggest that midtrimester ultrasound biometry or less restrictive diagnostic thresholds may help identify pregnancies with mild glucose intolerance at highest risk for fetal overgrowth.
